# Three-dimensionally fused gadolinium-enhanced and diffusion-weighted images: value in determination of multi-centricity of breast carcinoma

**DOI:** 10.1186/1470-7330-14-S1-S8

**Published:** 2014-10-09

**Authors:** H Sherif, A Mahfouz, A Kambal, A Sayedin, I Mujeeb

**Affiliations:** 1 Hamad Medical Corporation, Doha, Qatar

## Purpose

72 patients with biopsy-proven breast carcinoma were sent to MR imaging for preoperative assessment of multicentricity. Based on mastectomy/ lumpectomy specimens and further biopsies, 47 patients had single lesions, and 25 had multiple lesions with a total number of 116 proven malignant foci. The preoperative MR images were retrospectively reviewed and post-processed to obtain three-dimensional fused images of early gadolinium enhancement (encoded in red) and diffusion-weighted images (encoded in green) at b=1500 s/mm^2^. To eliminate the T2 shine-through effect, lesions with ADC ≥ 1× 10^−3^ mm ^2^ /s were eliminated. The post-processed images were reviewed by an experienced blinded radiologist, who noted all the lesions with a diameter ≥ 5 mm, classifying them into three groups: matched enhancement and diffusion restriction (matched E-DR), unmatched diffusion restriction (DR), and unmatched enhancement (E).

## Results

313 lesions with a diameter ≥ 5 mm were identified. 101 lesions showed matched E-DR. Taking matched E-DR as indicative of malignancy, sensitivity, specificity, positive predictive value, negative predictive value and accuracy for diagnosis of individual malignant foci were 84.5, 98.5, 97, 91, and 93.3 % respectively. Three false positive foci of matched E-DR were due to fibroadenomas. 18 false negative foci have been due to foci of DCIS less than 1 cm in diameter. Conclusion: Fused images of gadolinium enhancement and diffusion restriction offer a reasonably accurate assessment of multicentricity in patients with breast carcinoma.

